# Assessing Agreement When Agreement Is Hard to Assess—The Agatston Score for Coronary Calcification

**DOI:** 10.3390/diagnostics12122993

**Published:** 2022-11-29

**Authors:** Kristoffer Papsø Andersen, Oke Gerke

**Affiliations:** 1 Department of Clinical Research, University of Southern Denmark, 5000 Odense, Denmark; 2 Department of Nuclear Medicine, Odense University Hospital, 5000 Odense, Denmark

**Keywords:** agreement, Bland–Altman, calcium, computed tomography, difference plot, heart, method comparison, quantitative, repeatability, reproducibility

## Abstract

Method comparison studies comprised simple scatterplots of paired measurements, a 45-degree line as benchmark, and correlation coefficients up to the advent of Bland–Altman analysis in the 1980s. The Agatston score for coronary calcification is based on computed tomography of the heart, and it originated in 1990. A peculiarity of the Agatston score is the often-observed skewed distribution in screening populations. As the Agatston score has manifested itself in preventive cardiology, it is of interest to investigate how reproducibility of the Agatston score has been established. This review is based on literature findings indexed in MEDLINE/PubMed before 20 November 2021. Out of 503 identified articles, 49 papers were included in this review. Sample sizes were highly variable (10–9761), the main focus comprised intra- and interrater as well as intra- and interscanner variability assessments. Simple analysis tools such as scatterplots and correlation coefficients were successively supplemented by first difference, later Bland–Altman plots; however, only very few publications were capable of deriving Limits of Agreement that fit the observed data visually in a convincing way. Moreover, several attempts have been made in the recent past to improve the analysis and reporting of method comparison studies. These warrant increased attention in the future.

## 1. Introduction

The analysis of method comparison studies entered a new era with the seminal work of Douglas Altman and Martin Bland [1,2]. They extended Tukey’s difference plot [3], a scatterplot of differences of paired, quantitative measurements and their respective means, by the so-called *Limits of Agreement* (LoA). Assuming the paired differences to be roughly normally distributed, the LoA are equal to the mean of the differences plus/minus 1.96 the standard deviation of the differences, simply due to the empirical 68–95–99.7 rule (see, for instance, [4,5]). Thereby, the LoA represent an estimate for the limits within which 95% of all population differences lie. Bland–Altman analysis of agreement in method comparison studies was triggered by predominant presentations of scatterplots for the paired measurements against each other and correlation coefficients in the 1970s and 1980s which supposedly were insufficient for a proper assessment of agreement [1,2].

The cardiologist Arthur Agatston and his colleagues proposed a score for measuring coronary artery calcium in noninvasive cardiac diagnostics back in 1990 [6]. Based on a coronary computed tomography scan, the Agatston score is the total calcium score across all calcific lesions that are detected on slices obtained from the proximal coronary arteries. The Agatston score is today a cardiovascular risk factor in preventive medicine [7], and norm curves have been derived recently based on data of a Danish screening population in 50–75-year-old participants [8].

As any biomarker for disease, the Agatston score must be reproducible in varying settings, be it across different raters, scanners, or time points. A peculiarity of the nonnegative, integer Agatston score is its often-observed right-skewed distribution in cohorts that are free of clinical cardiovascular disease [9]. This impedes any agreement assessment of this score as otherwise widely applied Bland–Altman LoA do not immediately apply.

This review set out to answer the following three questions:

1. Which statistical measures and analyses were employed for agreement assessment of the Agatston score during the past three decades?
2. To which extent did the increased awareness of alternative analysis strategies (such as Bland–Altman LoA and related research) influence and change the way agreement assessments of the Agatston score were performed?
3. In light of recent research endeavors, what are potential routes for agreement analyses for highly skewed biomarkers like the Agatston score?

In the following, we will see:

- The material that agreement assessment was based on varied massively from a few dozens to thousands of observations.
- Simple scatterplots of paired measurements against each other and accompanying correlation coefficients have prevailed across three decades.
- Logarithmic transformations have been popular to counteract non-normally distributed Agatston scores.
- Bland–Altman analysis has been increasingly used, but in many variations.
- Only very few publications were capable of deriving LoA that fit the observed data nicely in a difference plot.

## 2. Materials and Methods

We consulted the most recently updated guideline for reporting systematic reviews and meta-analysis, PRISMA 2020 [10].

### 2.1. Literature SEARCH

We searched MEDLINE/PubMed for potentially relevant original articles in English on coronary calcification, agreement, and rater with the following search string on 20 November 2021:

(((agatston) OR (calcium AND score)) OR (coronary AND artery AND calcification)) AND (((((((agreement) OR (reliability)) OR (reliable)) OR (reproducibility)) OR (reproducible)) OR (repeatability)) OR (repeatable))) AND ((rater) OR (observer)).

We identified additional articles by screening the references of identified items. We excluded cases, case series, commentaries, editorials, opinions, preclinical studies, and reviews.

### 2.2. Data Extraction

One author (K.P.A.) screened titles, abstracts, and full texts and drafted the extraction of data. These included: first author, year of publication, type of agreement assessment (e.g., interrater, intra-rater, and inter-scan), type of scanner (electron beam computed tomography (EBCT), dual-slice helical computed tomography (DSCT), multi-slice computed tomography (MSCT)), details on Agatston score computation, scanner protocols, patient characteristics, number of observations, and statistical analysis. The other author (O.G.) reviewed and revised the extracted data.

### 2.3. Data Analysis

Descriptive statistics comprised median (minimum–maximum) for continuous and frequencies with respected percentages for categorical variables. Graphical displays consisted of boxplots. For all analyses, we used STATA/MP 17.0 (StataCorp, College Station, TX 77845, USA).

## 3. Results

**Figure 1:**
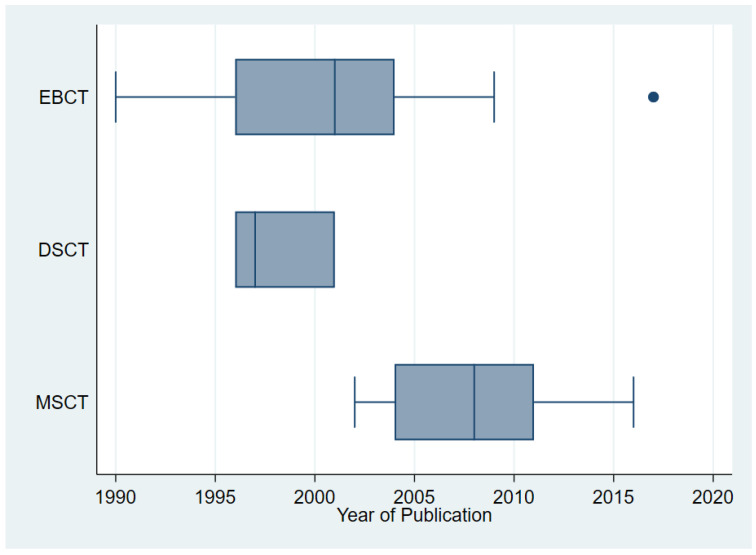
Type of CT scanner technology used in the included studies.

**Figure A1:**
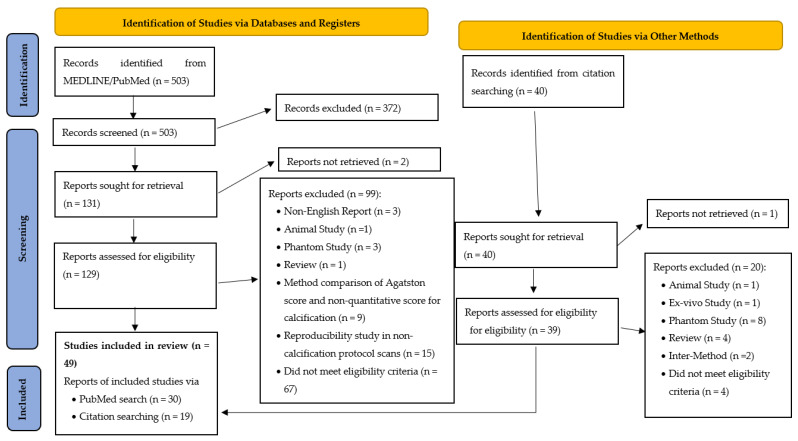
PRISMA flowchart [10].

The literature search resulted in 503 identified records from MEDLINE/PubMed and 40 additional records from searching citations (Figure A1). After screening of titles and abstracts and after full-text assessment, 49 studies remained [6,11,12,13,14,15,16,17,18,19,20,21,22,23,24,25,26,27,28,29,30,31,32,33,34,35,36,37,38,39,40,41,42,43,44,45,46,47,48,49,50,51,52,53,54,55,56,57,58]. These were published from 1990 to 2017 and used EBCT (*n* = 28), DSCT (*n* = 5), and MSCT (*n* = 23). Figure 1 visualizes the transition from EBCT in the 1990’s and early 2000’s to MSCT from 2004 on in the included studies.

The studies served reproducibility assessment with varying focus. In 26 studies, it was interrater variability (how well agree two raters assessing the same scans), in 16 studies, it was intra-rater variability (how well can a single rater reproduce earlier findings). Interscan (or, intra-scanner) reproducibility (how well agree consecutive scans of the same scanner) was investigated in 33 studies, interscanner reproducibility (how well agree scans of two different scanners) in five studies. One study [23] compared three acquisition protocols in DSCT.

**Figure 2:**
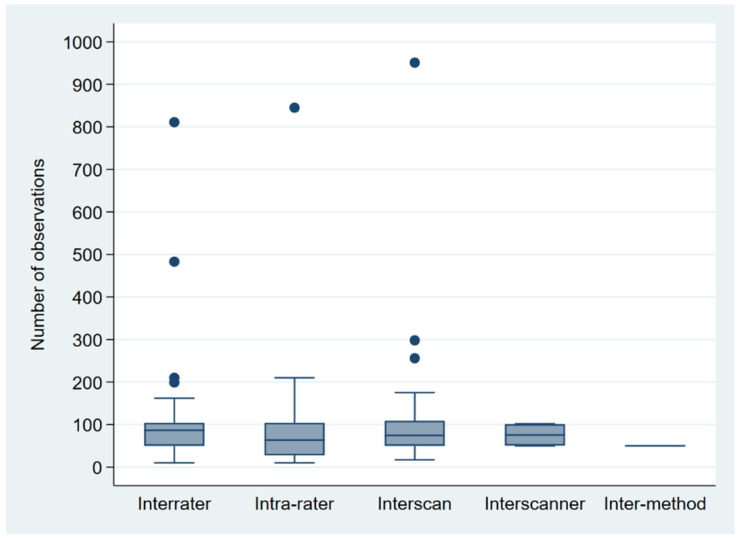
Boxplots for sample size by type of reproducibility assessment, excluding studies with more than 1000 observations [37,40,41,44,58] for better comparability.

Sample sizes varied between 10 [47] and 9761 [41], with a median of 85. Three-quarters of studies used 120 observations or fewer; 90% of the studies used 811 observations or fewer. Five studies employed more than 1000 observations [37,40,41,44,58]. Figure 2 shows boxplots for the sample size by type of reproducibility assessment, excluding studies with more than 1000 observations for better comparability. Including studies with more than 1000 observations, the third quartile was below 105 for all types of reproducibility assessment, apart from interscan (i.e., intra-scanner) variability (*n* = 175).

Table 1 summarizes the applied statistical tools of the included studies.

**Figure 3:**
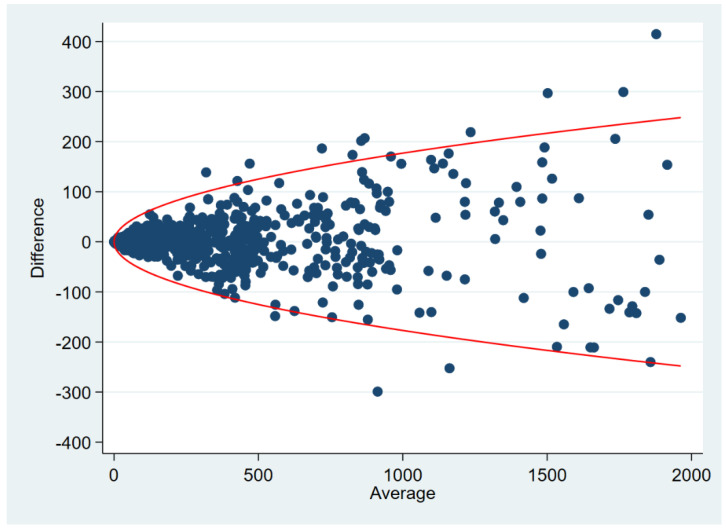
Plot shows a fictive distribution of differences between repeated Agatston scores. Curve shows 95% repeatability limits based on nonparametric quantile regression as applied in [40,58].

We observed Bland–Altman-like displays in every second study. These were scatterplots of means against absolute differences [16] or relative differences (in %) [18,22,24,29,31] as well as Tukey difference plots [11,23,44,48]. None of these contained any LoA. Since 2003, the reporting of LoA have become more common, but respective 95% confidence intervals (95% CI) for the LoA were refrained from [36,39,43,47,49,50,52,53,55,56,57]. Only four studies presented 95% CIs: one study did so for the LoA [54], one study applied a square root transformation to derive a 95% CI for the mean difference [37], and two studies [40,58] employed nonparametric quantile regression to assess 95% repeatability limits that form a parable opening up with increasing mean values of the Agatston score (Figure 3). Both studies [40,58] used fractional polynomial regression and tested several transformations of the Agatston score (i.e., square root, log, quadratic, etc.). In both studies, the optimal method was the square root transformation that then was used in a quantile regression model, and the response was the conditional 95th percentile of the absolute value of the difference between repeated measurements.

Some research groups chose to stick to simple LoA despite visually prevailing non-constant bias [50,52] or variance heterogeneity [47,52,53]. Others derived LoA that seemed to somewhat fit the data [36,39,55,56,57], partly working on logarithm-transformed data.

Nearly half of the studies analyzed relative changes (in %), and 20 out of 49 studies (41%) reported scatterplots of paired measurements (e.g., rater 1’s scores on the x-axis, rater 2’s scores on the y-axis), supplemented with Bravais-Pearson or Spearman’s rank correlation coefficients. At times, colleagues used linear regression to evaluate intercept and slope in the scatterplots of paired measurements to compare these with the 45-degree line of perfect agreement (10 out of 49 studies, 20%). One-third of the studies employed logarithmic transformations to account for the skewed distribution of Agatston scores. ANOVA (*n* = 13, 27%) and t tests (*n* = 10, 20%) were used for intergroup comparisons of mean values, intra-class coefficients (*n* = 10, 20%) and kappa (*n* = 6, 12%) served reliability assessment.

Every fourth study made use of proportions of agreement. Agatston et al. [6] reported identical scores of two raters in 70 out of 88 patients (79.5%), Kajinami et al. [11] indicated disagreement between three raters in 18 out of 75 cases (24%), and Lawler et al. [38] gave proportions for discordant pairs of measurement. Others reported proportions of agreement for Agatston scores categorized as 0 vs. >0 [19,41,43,44,50,58] and ≤10 vs. >10 [16], percentage of >25% [33] and >15% variability [47].

## 4. Discussion

### 4.1. Key Findings

- Sample sizes of studies assessing agreement of the Agatston score ranged from 10 to 9761, with a median of 85 and a third quartile of 120.
- Simple scatterplots of paired measurements against each other and accompanying correlation coefficients have prevailed across three decades.
- Logarithmic transformations have been popular to counteract skewed distributions of the Agatston score.
- Bland–Altman plots have replaced difference plots of various kinds, but 95% CIs accompanied the LoA only in 4 out of 49 studies (8%).
- Only two publications (4%) applied nonparametric quantile regression to derive 95% repeatability limits that fit the observed data nicely in a difference plot.

### 4.2. Limitations of the Study

The literature search was conducted in one database only, and the search string was optimized ad hoc without using any MeSH terms. Title and abstract screening were performed by only one author.

This study focused on the Agatston score for coronary calcification due to its prominent role in risk modelling [7]; however, other calcium scoring variables are calcium volume and calcium mass [20,59]. Moreover, our study investigated the various statistical analysis strategies used in the assessment of repeatability and reproducibility. As such, we excluded investigations into the effects of, say, heart rate, body mass index, and noise level on interscan and interrater variability of the Agatston score [59].

We neither examined the role of the Agatston score in coronary risk stratification, nor did we investigate its prognostic use in asymptomatic or symptomatic patients [60]. The American College of Cardiology Foundation Clinical Expert Consensus Task Force meta-analyzed data of 27,622 asymptomatic patients who participated in six large studies [61]. The authors pointed to an increasing relative risk of major cardiovascular events for patients with Agatston scores classified as 100–400, 401–999, and 1000 or more, compared to patients with an Agatston score of zero. Mickley et al. [62] investigated the diagnostic and short-term role of coronary computed tomography angiography-derived fractional flow reserve (FFR_CT_) in chest pain patients with an Agatston score exceeding 399, and Diederichsen et al. [63] employed Agatston score categories of 0, 1–399, and 400 and larger. Intra- and interrater variability of the categorized Agatston score is, naturally, small due to the limited number of categories [64], in opposition to analogous investigations of the continuous Agatston score (Figure 3) [8,40,58]. While we focused on repeatability and reproducibility assessments of the continuous Agatston score, it is widely used in clinical practice in a categorized fashion, thereby palliating its comparably large variability as continuous marker.

Shen et al. [65] described the natural course of Agatston score progression after 5 years in an Asian population with an initial score of zero. Spotty microcalcifications are below the resolution of computed tomography due to limited spatial resolution and develop in the earliest stages of coronary intimal calcification [66]. An Agatston score of zero may very well disguise microcalcification made visible by intravascular ultrasound or optical coherence tomography [66] or positron emission tomography/computed tomography [67,68]. These were, though, beyond the scope of this review.

Finally, we only considered the repeatability and reproducibility of the continuous Agatston score as stand-alone marker. Wu et al. [69] developed a prediction nomogram for Agatston score progression in an Asian population, based on a least absolute shrinkage and selection operator-derived logistic model. Building, validating, and applying medical risk prediction models, in which the Agatston score plays part, were also beyond the scope of the review. For further reading on this matter, we point the interested reader to the recently published textbook *Medical Risk Prediction Models* by Gerds and Kattan [70].

### 4.3. What This Adds to What Is Known

Kottner et al. [71] published the Guidelines for Reporting Reliability and Agreement Studies in 2011. They asserted the following: “Studies may be conducted with the primary focus on reliability and agreement estimation itself, or they may be a part of larger diagnostic accuracy studies, clinical trials, or epidemiological surveys. In the latter case, researchers report agreement and reliability as a quality control, either before the main study or by using data of the main study. Typically, results are reported in just a few sentences, and there is usually only limited space for reporting.” The variable sample size in our study clearly supports the notion that only larger diagnostic trials that evaluate agreement as a secondary endpoint were capable of providing results based on substantial materials consisting of more than 1000 observations. Examples of such larger trials are the Multi-Ethnic Study of Atherosclerosis [41,44] and the Dallas Heart Study [58].

The implementation of changes in current practice can be tedious [72,73]. Simple analysis tools such as scatterplots of raw measurements and accompanying correlation coefficients still enjoy great popularity though Bland–Altman analysis prevails as primary analysis from the early 2000s onwards. Bland and Altman’s careful indication of the necessity of 95% CI for the LoA in their seminal paper published in 1986 (“We might sometimes wish to use standard errors and confidence intervals to see how precise our estimates are, provided the differences follow a distribution which is approximately Normal.” [2]) got cut short as per editorial request. Consequently, 95% CIs for the LoA were often missing [74]. Apparently, the only study providing 95% CIs for the LoA [54] did so by using approximate 95% CIs as originally suggested [2,75] although improvements have been suggested continuously [76,77,78,79,80,81,82].

Likewise, several research groups pinpointed repeatedly that scatterplots of raw measurements are insufficient for agreement assessments and adjustments to Bland–Altman analysis are necessary in the case of non-constant bias and variance heterogeneity [75,83,84,85,86]. For instance, Bland and Altman [83] discussed comprehensively both the interpretation of correlation coefficients and regression lines. Issues with focusing the analysis strategy on correlation coefficients are twofold: Firstly, the correlation depends on how the subjects were sampled as both the width of the measurements range and the distribution of the two variables influence the correlation between two variables. Secondly, correlation assesses the degree of association, not agreement. Perfect agreement requires good correlation, but good correlation is not sufficient for good agreement as measurements in a scatterplot may roughly scatter around a line that is parallel to the 45-degree line.

Bland and Altman [83] did also discuss and exemplify why simple linear regression of one method’s measurements on the other method’s measurements is inappropriate in method comparison studies. The regression attempts to predict the observed values Y of one method from the observed values X of the other method, not the true Y from the true X. The measurement errors in X reduce the slope of the regression line so that the lower end of the regression line is raised and the upper end is lowered. Because of this, the value of the intercept exceeds zero. Consequently, hypothesis tests on the intercept equaling 0 and the slope equaling 1 are misleading.

Method comparison studies in clinical chemistry often employ Deming regression that is a technique for fitting a regression line through a scatterplot of raw measurements of two variables that are measured with error [87,88,89]. Deming regression is often used to look for systematic differences between two measurement methods. Another approach in that area is Passing–Bablok regression [90,91,92].

### 4.4. What Is the Implication, What Should Change Now?

There have been several attempts to suggest reporting standards for Bland–Altman analysis to harmonize analysis and reported outcomes [93,94]. Bland and Altman [75,83] reckoned that the LoA approach is fundamentally very simple and direct. It requires assumptions of constant bias and homogeneity of the variances across the measurement range, but the LoA are easy to interpret in relation to pre-specified clinical benchmarks for clinically sufficient agreement between methods. The LoA approach can be extended to situations that are more complex when assumptions are not met (e.g., non-constant bias or increasing variance with increasing measurements), when there are repeated measurements on the same subject, and when there are varying numbers of observations on subjects. Bland and Altman [75] even proposed a nonparametric version of the LoA instead of which several nonparametric estimators such as the Harrell–Davis estimator and estimators of the Sfakianakis–Verginis type can be used also [95].

Recently, Taffé [96,97,98,99,100] as well as Chen and Kao [101] proposed alternative routes for agreement assessment. Taffé [100] proposed a set of bias, precision, and agreement plots whenever an assumption of constant bias or homogeneity of the variances does not hold. His approach does though require repeated measurements by at least one of the two measurement methods. Chen and Kao [101] proposed a more general framework beyond the difference-based LoA approach. They proposed two analysis strategies: With known distributions, they generalized Bland–Altman LoA by a parametric approach, leading to a general measure of closeness which allows the researcher to tackle situations with more general distributions. With unknown distributions, Chen and Kao proposed a nonparametric approach using quantile regression, leading to an assessment of agreement without distributional assumptions. They reckon that both approaches account for systematic and random measurement errors.

Sample size recommendations have been comparably scarce [71,82,102,103] which is likely due to the common view that agreement assessment is an estimation, not a hypothesis testing problem. Still, the accuracy in terms of the expected width of 95% CIs for the LoA may serve as a basis for a sample size rationale. Moreover, most agreement assessments are ‘just’ a spin-off to a larger investigation, thereby putting some restrictions on the possible sample size for agreement assessment. Admittedly, agreement assessments for the Agatston score span widely, comprising intra- and interrater as well as intra- and interscanner variability. The extent of these possible variability targets inflates the extent of possible and necessary analyses when investigating several items in one study (intra- and interrater repeatability as well as intrascanner variability). However, several attempts have been made in the past to improve the analysis and reporting of agreement studies [71,75,78,79,80,82,84,86,93,94,95,96,97,98,99,100,101].

There is still room for improvement on the way to appropriately deriving 95% repeatability limits that nicely fit the observed paired data [40,54,58,75,86] or, alternatively, to follow alternative analysis routes [96,97,98,99,100,101]. The most appropriate analysis strategy will always depend on the observed distribution of the paired differences at hand. Personalized precision medicine and inherent risk scores in future noninvasive medicine that may help to identify patients at greatest risk of cardiovascular disease and its complications require the validity and reproducibility of any prediction marker or model.

## Figures and Tables

**Table 1:** Distribution of applied statistical techniques in the included studies (*n* = 49).

Type of Analysis	Number of Studies (%)
Bland–Altman plot or a variant thereof ^1^	25 (51)
Relative change (in %)	23 (47)
Scatterplot of paired measurements	20 (41)
Correlation coefficient	20 (41)
Logarithmic transformation	15 (31)
ANOVA	13 (27)
Proportion of agreement	12 (25)
*T* test	10 (20)
Linear regression	10 (20)
Intra-class correlation coefficient	10 (20)
Kappa	6 (12)
Quantile regression	2 (4)

^1^ Display of differences or, alternatively, percentage changes against mean values.

## Data Availability

Not applicable.

## Appendix A

The appendix contains the PRISMA flowchart [10].
